# Diagnostic image quality of shoulder magnetic resonance imaging at 0.55T in healthy subjects and patients with rotator cuff injury: Prospective comparison with 1.5T

**DOI:** 10.1002/acm2.70825

**Published:** 2026-10-20

**Authors:** Jing Liu, Yue Gao, Ge Gao, Wei Li, Xiang Wang, Jinxia Zhu, Xiaoming Liu, Xiaoye Wang, Kun Zhou, Heng Liu, Jianxing Qiu

**Affiliations:** ^1^ Department of Radiology Peking University First Hospital Beijing China; ^2^ MR Collaboration Siemens Healthcare, Ltd. Beijing China; ^3^ MR Clinical Marketing Siemens Healthineers Beijing China; ^4^ Siemens Shenzhen Magnetic Resonance Ltd. Shenzhen China; ^5^ Department of Orthopedics Peking University First Hospital Beijing China

**Keywords:** deep learning (DL), flexible position, image quality, magnetic resonance imaging (MRI), shoulder imaging

## Abstract

**Background:**

Lower‑field 0.55 T MRI scanners with large‑bore design could offer patients more comfortable feeling and flexible position. With the advances in deep‑learning reconstruction and hardware optimization, 0.55T have greatly improved their image quality and cost‑effectiveness for musculoskeletal applications. However, its feasibility for shoulder evaluation remains unstudied.

**Purpose:**

To evaluate the image quality of shoulder using 0.55T MRI in volunteers and patients with rotator cuff injury with comparison of conventional 1.5T.

**Methods:**

Fifty participants (25 volunteers, 25 patients) were prospectively enrolled. All participants underwent shoulder MRI both at open 0.55T with contour coils and deep learning (DL) and 1.5T MR without DL. The nonparametric method was utilized to compare image quality scores and quantitative metrics (signal‐to‐noise ratio (SNR) and contrast‐to‐noise ratio (|CNR|)). Arthroscopy have been conducted in 16 patients. Kappa statistics was used to assess interobserver and interstudy agreement for lesion evaluation. Comfort feeling was also compared.

**Results:**

The scan time was 11′25″ for 0.55T MRI and 11′42″ for 1.5T MRI. The interobserver analyses showed high consistency (κ = 0.771–1.000, *P: 0.008 ∼ *< 0.001) among 3 readers. The scores for coronal fat suppressed T2 weighted imaging (fsT2WI) and coronal T1 weighted imaging (T1WI) were significantly higher for 0.55T MRI. Most sequences at 0.55T demonstrated significantly higher SNRs (*P:0.04 ∼ *< 0.001). Additionally, |CNR| values for lesions were significantly higher in fs proton weighted imaging (PDWI) and fsT2WI at 0.55T. Perfect interstudy agreement between 0.55T and 1.5T was observed between 2 readers (Kappa = 1, *P *< 0.001) for lesion assessment. Agreement between MRI findings and arthroscopic results was perfect or good. The 0.55T system received higher scores in comfort ratings.

**Conclusion:**

The 0.55T MRI with DL, showed comparable or better image quality in healthy subjects and patients with rotator cuff injury to 1.5T MRI without DL.

## 1 INTRODUCTION

Due to increased space and reduced noise, lower‐field magnetic resonance imaging (MRI) systems (0.55T) with larger bore sizes have gained more clinical adoption in various applications, including brain, fetal, and lung imaging.^1^, ^2^, ^3^, ^4^, ^5^, ^6^ Previous studies have demonstrated that image quality at 0.55T is comparable to that at 1.5T; in some domains, it is comparable to the quality at 3T.^7^, ^8^, ^9^, ^10^

Diagnostic image quality has also been reported for musculoskeletal imaging at 0.55T, including the knee and hip.^4^, ^8^, ^11^ Although higher magnetic field strength generally provides superior anatomical details, hardware advances such as coils and artificial intelligence‐assisted imaging optimization have enabled 0.55T MRI scanners to demonstrate robust clinical potential.^12^, ^13^ Dedicated joint coil systems yield higher signal‐to‐noise ratios (SNRs) while enhancing patient comfort, particularly in joints’ examinations, such as knee.^4^ Image reconstruction techniques based on deep learning (DL) algorithms improve the limitations related to acquisition speed and SNR.^14^, ^15^ Additionally, enhanced magnet efficiency and reduced helium consumption contribute to the cost‐effectiveness of 0.55T MRI systems.^13^

However, as so far, no studies have explored the feasibility of shoulder imaging using low‐field MRI, including 0.55T systems. MRI is recognized for its high contrast resolution that enables clear visualization of bone marrow, cartilage, ligaments, and soft tissue, underscoring its importance in shoulder evaluation.^14^, ^15^, ^16^ Since shoulder is one of the broadest anatomical regions of body, conventional MRI systems with a 70‐cm bore may not adequately accommodate patients with obesity. An MRI scanner with a larger aperture—such as the 0.55T system with an open 80‐cm bore—may provide a more comfortable experience for shoulder assessment. Furthermore, greater flexibility in shoulder positioning can be achieved at 0.55T, potentially allowing improved visualization of labral tears and tendon lesions.

Considering that previous studies have demonstrated excellent performance of DL algorithms in knee joint imaging at 0.55T,^4^, ^8^, ^17^ the present study aimed to investigate 2D shoulder MRI sequences performed in both neutral and abducted positions. Image quality and diagnostic performance at 0.55T were evaluated in comparison with a 1.5T MRI system. Additionally, participant experience and satisfaction between the two modalities were assessed.

## 2 MATERIALS AND METHODS

### 2.1 Study design and eligibility criteria

This prospective, single‐center study was conducted in accordance with the Declaration of Helsinki and was approved by the institutional review board of local Hospital. Written informed consent was obtained from all participants, including both volunteers and patients.

The sample size for this study was calculated based on a paired design comparing mean differences with the formula as following:

$$n=\frac{{\left(z_{\alpha }+z_{\beta }\right)}^{2}*\sigma^{2}}{\delta^{2}}$$

The Type I error probability was set at 0.05 (two‐tailed), and the specified statistical power (1 ‐ β) was set as 0.9. With results of pilot study, we set the expected mean difference (δ) between the paired measurements of SNR as 15, and the estimated standard deviation of differences (σ) as 30. Thus, we got the estimated sample size as 43.

In total, 50 participants (25 volunteers and 25 patients) were enrolled. Volunteer group: In July 2024, 25 healthy volunteers underwent shoulder MRI examinations at both 0.55T and 1.5T. Among these, 23 underwent right shoulder imaging and two underwent left shoulder imaging (the scanning shoulder was selected subjectively by the participants). Each volunteer also underwent a shoulder examination in a 45° abducted position on the 0.55T system. A satisfaction survey was conducted in this cohort.

Patient group: From August to November 2024, 25 consecutive patients with suspected rotator cuff injuries by clinician were enrolled to undergo shoulder MRI examinations at both 0.55T and 1.5T. Among these, 19 underwent right shoulder imaging and six underwent left shoulder imaging. Rotator cuff injury occupies largely in arthroscopy surgery in our general hospital, the inclusion criteria for this study were (1) former shoulder imaging indicating rotator cuff injuries and (2) shoulder pain with weak of specific muscle and suspected rotator cuff injury.

Imaging quality was compared in both cohorts, and lesion evaluation in the patient group was performed using arthroscopy as the reference standard. Exclusion criteria were (1) contraindications to MRI (e.g., cardiac pacemaker, claustrophobia) and (2) pregnancy. The flowchart is presented in Figure 1.

**FIGURE 1:**
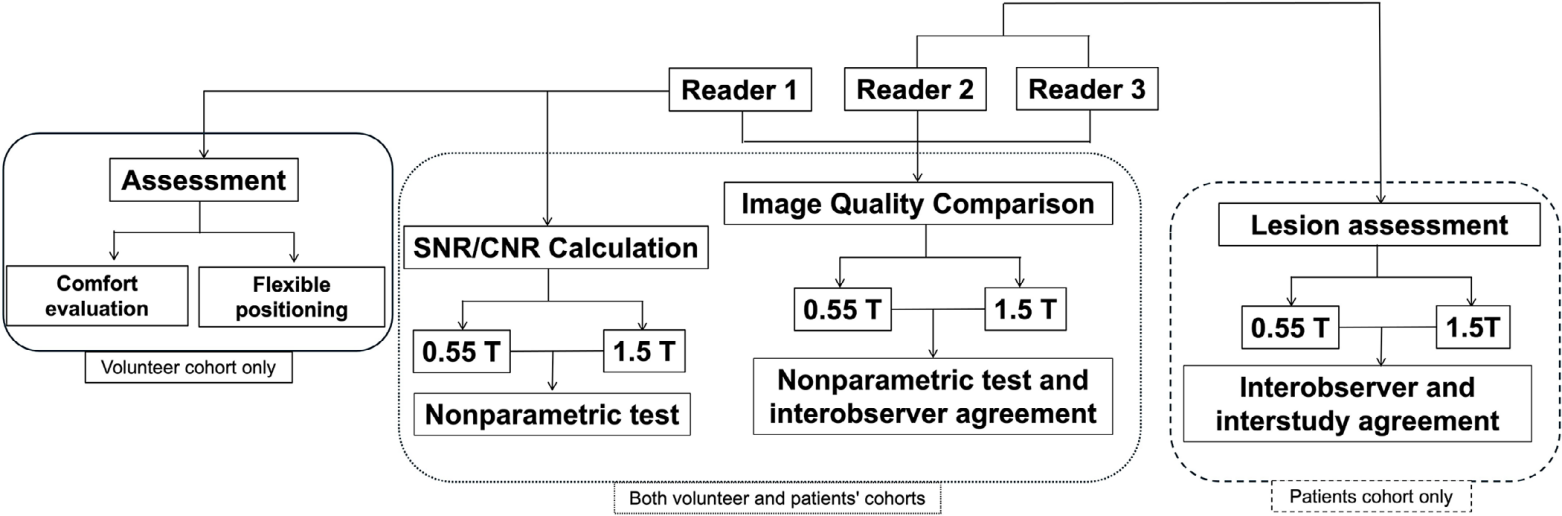
Workflow diagram SNR: signal‐to‐noise ratio, CNR: contrast‐to‐noise ratio.

### 2.2 MRI examinations

All MRI examinations were performed using 0.55T and 1.5T whole‐body scanners (MAGNETOM Free Max (software version: XA60) and MAGNETOM Aera (software version: VE11C), Siemens Healthcare, Erlangen, Germany). The 0.55T scanner was equipped with a flexible 12‐channel contour coil, whereas the 1.5T scanner utilized an 18‐channel phased‐array transmit/receive shoulder coil (Siemens Healthcare, Erlangen, Germany). The bore size was 80 cm*148 cm, 70 cm*135 cm (width*length) for 0.55T and 1.5T, respectively. Images acquired at 0.55T were reconstructed using a DL algorithm.

In the volunteer cohort, the MRI protocol included fat‐suppressed (fs) proton density‐weighted imaging (PDWI) in the coronal (COR) and axial (AX) planes, as well as COR T1‐weighted imaging (T1WI) and COR fsT2‐weighted imaging (fsT2WI). Additionally, each volunteer underwent COR fsPDWI at 0.55T in a 45° abducted shoulder position to evaluate the feasibility of flexible positioning. All volunteers completed both MRI examinations within a 2‐week period; 1.5T scanning was conducted prior to 0.55T scanning.

In the patient cohort, the MRI protocol included AX T1WI, AX fsPDWI, COR T1WI, COR fsPDWI, COR fsT2WI, and sagittal (SAG) fsPDWI. All patients completed both MRI examinations on the same day; 0.55T scanning was performed before 1.5T scanning.

Sequence parameters for both 0.55T and 1.5T in the patient cohort are summarized in Supplementary 1, and results of phantom experiment study are shown in Supplementary 2 and Supplementary 3.

### 2.3 DL‐Based Reconstruction Algorithm

A DL‐based reconstruction pipeline, Deep Resolve Boost (DRB; Siemens Healthineers, Forchheim, Germany),^4^, ^18^ was employed at 0.55T in this study. The DRB method utilized convolutional neural networks (CNNs)^15^, ^19^ to incorporate both specific noise maps and original raw data during image reconstruction. CNNs were trained on large datasets to learn robust transformation mappings from sensor space to the image domain. DRB enhanced the SNR and enabled high‐quality image reconstruction at 0.55T. Additionally, simultaneous multi‐slice imaging was performed at 0.55T to concurrently excite and acquire multiple slices, thereby accelerating the process and reducing acquisition time.

### 2.4 Qualitative assessment

Qualitative assessment was performed in both cohorts. Three radiologists (AA, BB, and CC, with 17, 5, and 3 years of experience in general radiology, respectively) independently evaluated image quality without prior knowledge of the scanning protocol, radiologic reports, and evaluation results from other readers. The image assessment was carried out in a random order, 20–25 examinations were read by each reader every day. To reduce recall bias, two examinations for the same volunteer/patient were separated to read with interval of two weeks above. All the images could be viewed without participant‐ or parameters‐related information.

Image quality was rated using a 5‐point Likert scale as follows:

1. Non‐diagnostic for poor image quality: Severe artifacts, image distortion, or inadequate signal intensity; no diagnostic confidence.
2. Moderate image quality: Presence of moderate artifacts and image distortion; limited diagnostic confidence.
3. Relatively good diagnostic image quality: Minimal artifacts and slight distortion; main structures reasonably well visualized; moderate diagnostic confidence.
4. Good diagnostic image quality: Few artifacts and slight distortion; clear visualization of structures; high diagnostic confidence.
5. Excellent image quality: Minimal to no artifacts or distortion; excellent depiction of bones, glenoid labrum, and muscle tendons; strong diagnostic confidence.

Image quality scores were recorded independently by the three readers in a blinded and randomized order. Average scores were compared between 0.55T and 1.5T. Interobserver agreement for image quality evaluation was also assessed.

### 2.5 Quantitative assessment

Quantitative assessment included evaluations of SNR and contrast‐to‐noise ratio (CNR). In both cohorts, SNR values for bone marrow and muscle were measured at both 0.55T and 1.5T MRI. In the volunteer cohort, the absolute value of the CNR (|CNR|) for bone marrow was also assessed. In the patient cohort, the |CNR| of lesions was evaluated.

Signal intensity (SI) was measured as follows:

For both cohorts, a round region of interest (ROI) with an area of approximately 200 mm^2^ was placed on the humeral head, avoiding any edematous regions, to measure the SI of bone marrow. A similarly sized ROI was placed on the deltoid muscle, also avoiding edematous regions, to measure muscle SI. A third ROI, placed in air distant from the shoulder, was used to determine the standard deviation (SD) of background noise for each sequence.

In the patient cohort, an additional round ROI with an area of approximately 20 mm^2^ was placed on the supraspinatus tendon to determine the SI of the lesion.

SNR and |CNR| values for bone marrow, muscle, and lesions were calculated using the following formulas:

$$\mathrm{SNR}=SI_{\text{(bone marrow/muscle)}}/SD_{\mathrm{noise}}$$

$${\left|\mathrm{CNR}\right|}_{\text{for volunteers}}=\left|\left(SI_{\text{(bone marrow)}}-SI_{\mathrm{muscle}}\right)\right|/SD_{\mathrm{noise}}$$

$$\begin{array}{cc} & {\left|\mathrm{CNR}\right|}_{\text{for lesion}}\\ & \;=\left|\;\left(SI_{\text{(supraspinatus tendon)}}-\;SI_{\mathrm{muscle}}\right)\right|\;/\;SD_{\mathrm{noise}} \end{array}$$

### 2.6 Diagnostic performance

Lesion evaluation included assessment of bone marrow edema in the humeral head, glenoid labrum tears, tendon injuries (supraspinatus, infraspinatus, biceps, subscapularis), ligament injuries (glenohumeral ligament, coracoacromial ligament, coracoclavicular ligament, and coracohumeral ligament), and joint effusion.

For the humeral head, Grades 0 and 1 represented absence and presence of bone edema, respectively.

For the glenoid labrum, Grades 0, 1 and 2 denoted normal appearance, labral injury, and labral tear, respectively.

For muscle tendons and ligaments, Grades 0, 1, 2, 3 and 4 indicated normal appearance, partial tear of I^0^ (0 < length of tear < 3 mm), partial tear of II^0^ (3 ≤ length of tear ≤ 6 mm), partial tear of III^0^ (length of tear > 6 mm) and complete tear, respectively.

For joint effusion, Grade 0 constituted normal, whereas Grade 1 denoted joint effusion.

Two radiologists (reader 2 and reader 3, with 5 and 17 years of experience in general radiology, respectively) independently evaluated the lesions in a blinded and randomized manner, as illustrated in Figure 1. Interobserver agreement between the two radiologists was examined, and interstudy agreement for lesion evaluation was calculated using reader 3, who had greater diagnostic experience, as the reference.

### 2.7 Volunteer comfort survey

After the two MRI examinations, volunteers completed a questionnaire evaluating five categories: (1) sense of space, (2) noise, (3) bed comfort, (4) coil comfort, and (5) overall impression. Each category was rated on a 5‐point Likert scale (1 = much worse, 5 = much better).

### 2.8 Statistical analysis

To avoid selection bias, consecutive patients were enrolled. Blind reading and standard measurements were used to avoid information bias. Statistical analysis was conducted using SPSS software (version 24.0; IBM, Chicago, IL). Kappa test was used to assess interobserver agreement for image quality scores. The Wilcoxon signed‐rank test, a nonparametric method, was utilized to compare image quality scores and quantitative metrics (SNR and |CNR|) between 0.55T and 1.5T MRI. For lesion evaluation, interobserver agreement and interstudy agreement between 0.55T and 1.5T MRI for each observer were assessed using kappa statistics. Mann‐Whitney U test was used to compare the comfort feeling scores.

## 3 RESULTS

### 3.1 Demographics

For 25 healthy volunteers, there were 12 men and13 women. The mean age was 26 ± 3 years (range: 23–30 years). The average BMI was 22.38 ± 4.35 kg/m^2^.

For 25 Patients: there were 10 men and 15 women. The mean age was 54 ± 2 years (range: 38–71 years). The average BMI was 25.57 ± 7.41 kg/m^2^.

### 3.2 Scan time

In the patient cohort, total scan times were 11 min 25 s for 0.55T MRI and 11 minutes 42 seconds for 1.5T MRI (Supplements 1).

### 3.3 Image quality

Qualitative scores were significantly higher for COR fsT2WI and COR T1WI at 0.55T compared with 1.5T. Other sequences demonstrated comparable image quality between the two modalities.

Perfect interobserver agreement was recorded for image quality in AX fsPDWI, COR fsT2WI, and COR T1WI at 0.55T (Kappa values ranging from 0.804 to 1.000, *P *< 0.05). Good to moderate agreement was noted for COR fsPDWI and AX T1WI (Table 1).

**TABLE 1:** Subjective image quality evaluation by Three readers.

							Interobserver agreement			
			Comparison between 0.55T and 1.5T MRI individually				0.55T		1.5T	
Sequence	Cohort	Reader	Scores for 0.55T	Scores for 1.5T	Z	*P*	Kappa	*P*	Kappa	*P*
Axial fsPDWI	Volunteers	1	4.71 ± 0.46	4.88 ± 0.34	−1.41	0.157	0.804	<0.001^*^	0.771	<0.001^*^
		2	4.67 ± 0.48	4.79 ± 0.51	−0.905	0.366				
		3	4.70 ± 0.46	4.88 ± 0.34	−1.41	0.157				
	Patients	1	4.88 ± 0.33	4.92 ± 0.28	−5.77	0.564	1.00	<0.001^*^	0.720	<0.001^*^
		2	4.88 ± 0.33	4.92 ± 0.28	−5.77	0.564				
		3	4.88 ± 0.33	4.84 ± 0.37	−0.447	0.655				
	Combined	1	4.80 ± 0.41	4.90 ± 0.31	−1.508	0.132	0.877	<0.001^*^	0.749	<0.001^*^
		2	4.78 ± 0.42	4.86 ± 0.41	−1.069	0.285				
		3	4.80 ± 0.41	4.86 ± 0.35	−0.832	0.405				
Coronal fsT2WI	Volunteers	1	5.00 ± 0.00	4.54 ± 0.51	−3.317	0.001^*^	1.00	<0.001^*^	0.798	<0.001^*^
		2	5.00 ± 0.00	4.63 ± 0.49	−3.00	0.003^*^				
		3	5.00 ± 0.00	4.75 ± 0.44	−2.449	0.014^*^				
	Patients	1	4.95 ± 0.21	4.63 ± 0.49	−2.646	0.008^*^	1.00	<0.001^*^	0.936	<0.001^*^
		2	4.95 ± 0.21	4.59 ± 0.50	−2.828	0.005^*^				
		3	4.95 ± 0.21	4.64 ± 0.49	−2.646	0.008^*^				
	Combined	1	4.98 ± 0.15	4.59 ± 0.50	−4.243	<0.001^*^	1.00	<0.001^*^	0.870	<0.001^*^
		2	4.98 ± 0.15	4.61 ± 0.49	−4.123	<0.001^*^				
		3	4.98 ± 0.15	4.70 ± 0.47	−3.606	<0.001^*^				
Coronal T1WI	Volunteers	1	4.88 ± 0.34	4.54 ± 0.59	−2.53	0.011^*^	0.859	<0.001^*^	0.890	<0.001^*^
		2	4.92 ± 0.28	4.63 ± 0.58	−2.646	0.008^*^				
		3	4.88 ± 0.34	4.54 ± 0.59	−2.530	0.011^*^				
	Patients	1	4.96 ± 0.20	4.72 ± 0.46	−2.121	0.034^*^	1.000	<0.001^*^	1.00	<0.001^*^
		2	4.96 ± 0.20	4.72 ± 0.46	−2.121	0.034^*^				
		3	4.96 ± 0.20	4.72 ± 0.46	−2.121	0.034^*^				
	Combined	1	4.92 ± 0.28	4.63 ± 0.53	−3.30	0.001^*^	0.902	<0.001^*^	0.940	<0.001^*^
		2	4.94 ± 0.24	4.67 ± 0.52	−3.357	0.001^*^				
		3	4.92 ± 0.28	4.63 ± 0.53	−3.30	0.001^*^				
Coronal fsPDWI	Volunteers	1	4.83 ± 0.38	4.58 ± 0.50	−1.732	0.083	0.619	<0.001^*^	0.943	<0.001^*^
		2	4.96 ± 0.20	4.54 ± 0.51	−2.887	0.004^*^				
		3	4.83 ± 0.38	4.58 ± 0.50	−1.732	0.083				
	Patients	1	4.96 ± 0.20	4.92 ± 0.28	−0.577	0.564	0.306	0.008^*^	0.842	<0.001^*^
		2	4.92 ± 0.28	4.88 ± 0.33	−0.447	0.655				
		3	5.00 ± 0.00	4.92 ± 0.28	−1.414	0.157				
	Combined	1	4.90 ± 0.31	4.76 ± 0.43	−1.807	0.071	0.546	<0.001^*^	0.929	<0.001^*^
		2	4.94 ± 0.24	4.71 ± 0.46	−2.668	0.008^*^				
		3	4.92 ± 0.28	4.76 ± 0.43	−2.138	0.033^*^				
Sagittal fsPDWI	Patients	1	4.72 ± 0.46	4.80 ± 0.41	−0.707	0.480	0.859	<0.001^*^	0.921	<0.001^*^
		2	4.80 ± 0.41	4.76 ± 0.44	−0.333	0.739				
		3	4.72 ± 0.46	4.80 ± 0.41	−0.707	0.480				
Axial T1WI	Patients	1	4.92 ± 0.28	4.80 ± 0.41	−1.134	0.257	0.786	<0.001	0.921	<0.001
		2	4.96 ± 0.20	4.80 ± 0.41	−1.633	0.102				
		3	4.92 ± 0.28	4.76 ± 0.44	−1.414	0.157				

* Statistically significant *P*‐value.

**Abbreviations**: PDWI, proton density‐weighted imaging; T1WI, T1‐weighted imaging; T2WI, T2‐weighted imaging.

### 3.4 Comparison of SNR and CNR

Most sequences at 0.55T demonstrated significantly higher SNRs in both patient and volunteer cohorts (*P *< 0.001) (Tables 2 and 3; Figure 2, 3). Additionally, |CNR| values for lesions were significantly higher in fsPDWI and fsT2WI sequences at 0.55T (*P *< 0.001) (Table 3; Figures 3, 4, 5, 6).

**TABLE 2:** Quantitative comparison of bone marrow and muscle between 0.55T and 1.5T MRI in volunteers across different sequences.

		SNR			
		0.55T	1.5T	*Z*	*P*
Axial fsPDWI	Bone marrow	43.44 ± 9.94	39.02 ± 17.59	−3.686	<0.001^*^
	Muscle	84.01 ± 21.73	80.49 ± 24.42	−1.916	0.055
Coronal fsPDWI	Bone marrow	64.39 ± 10.54	38.93 ± 25.86	−4.286	<0.001^*^
	Muscle	87.97 ± 29.12	61.21 ± 26.16	−3.886	<0.001^*^
Coronal T1WI	Bone marrow	342.29 ± 98.59	272.96 ± 86.19	−3.571	<0.001^*^
	Muscle	99.97 ± 26.46	75.96 ± 19.79	−4.057	<0.001^*^
Coronal fsT2WI	Bone marrow	60.93 ± 15.80	37.55 ± 19.40	−3.857	<0.001^*^
	Muscle	72.82 ± 23.10	46.42 ± 20.20	−4.200	<0.001^*^

* Statistically significant *P*‐value.

**Abbreviations**: PDWI, proton density‐weighted imaging; SNR, signal‐to‐noise ratio; T1WI, T1‐weighted imaging; T2WI, T2‐weighted imaging.

**TABLE 3:** Quantitative comparison of bone marrow and muscle between 0.55T and 1.5T MRI in patients across different sequences.

		SNR				|CNR|			
		0.55T	1.5T	*Z*	*P*	0.55T	1.5T	*Z*	*P*
Axial fsPDWI	Bone marrow	41.84 ± 11.80	34.84 ± 11.06	−2.570	0.01^*^	——			
	Muscle	74.64 ± 18.86	69.10 ± 60.45	−3.188	0.001^*^				
Axial T1WI	Bone marrow	339.58 ± 94.04	218.35 ± 79.44	−4.372	<0.001^*^	——			
	Muscle	107.16 ± 29.03	107.16 ± 29.03	−3.700	<0.001^*^				
Coronal	Bone marrow	61.10 ± 19.09	31.67 ± 13.40	−3.969	<0.001^*^	34.64 ± 19.55	26.70 ± 14.60	−3.484	<0.001^*^
fsPDWI									
	Muscle	132.52 ± 31.55	55.23 ± 27.67	−2.704	0.007^*^				
Coronal	Bone marrow	342.50 ± 119.73	204.60 ± 62.01	−4.292	<0.001^*^	——			
T1WI									
	Muscle	98.43 ± 30.99	61.42 ± 18.53	−4.319	<0.001^*^				
Sagittal	Bone marrow	24.51 ± 7.85	19.90 ± 6.67	−2.657	0.008^*^	26.27 ± 11.54	19.18 ± 9.50	−3.969	<0.001^*^
fsPDWI	Muscle	44.33 ± 14.26	40.65 ± 16.26	−2.058	0.040*				
Coronal	Bone marrow	42.06 ± 10.91	31.96 ± 9.48	−3.584	<0.001^*^	76.82 ± 43.40	43.81 ± 21.14	−3.920	<0.001^*^
fsT2WI									
	Muscle	51.58 ± 13.50	39.12 ± 9.34	−3.883	<0.001^*^				

* Statistically significant *P*‐value.

**Abbreviations**: |CNR|, absolute value of contrast‐to‐noise ratio; PDWI, proton density‐weighted imaging; SNR, signal‐to‐noise ratio; T1WI, T1‐weighted imaging; T2WI, T2‐weighted imaging.

**FIGURE 2:**
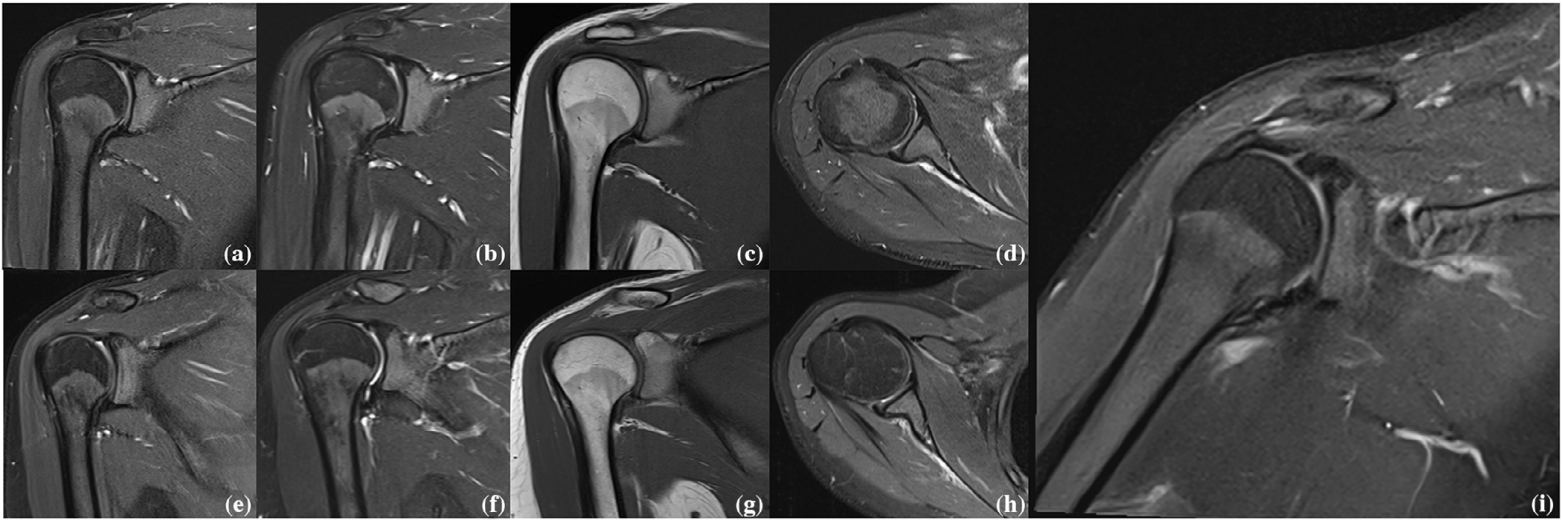
Image comparison between 0.55T and 1.5T in a volunteer. Shoulder MRI of a 23‐year‐old female volunteer. Images (a–d) show 0.55T scans: coronal (COR) fat‐suppressed proton density‐weighted imaging (fsPDWI), COR fat‐suppressed T2‐weighted imaging (fsT2WI), COR T1‐weighted imaging (T1WI), and axial (AX) fsPDWI, respectively. Images (e–h) show corresponding sequences acquired at 1.5T, matched to (a–d). All images from 0.55T demonstrated comparable quality to those from 1.5T. Image I shows the abducted position (45°) of COR fsPDWI acquired at 0.55T.

**FIGURE 3:**
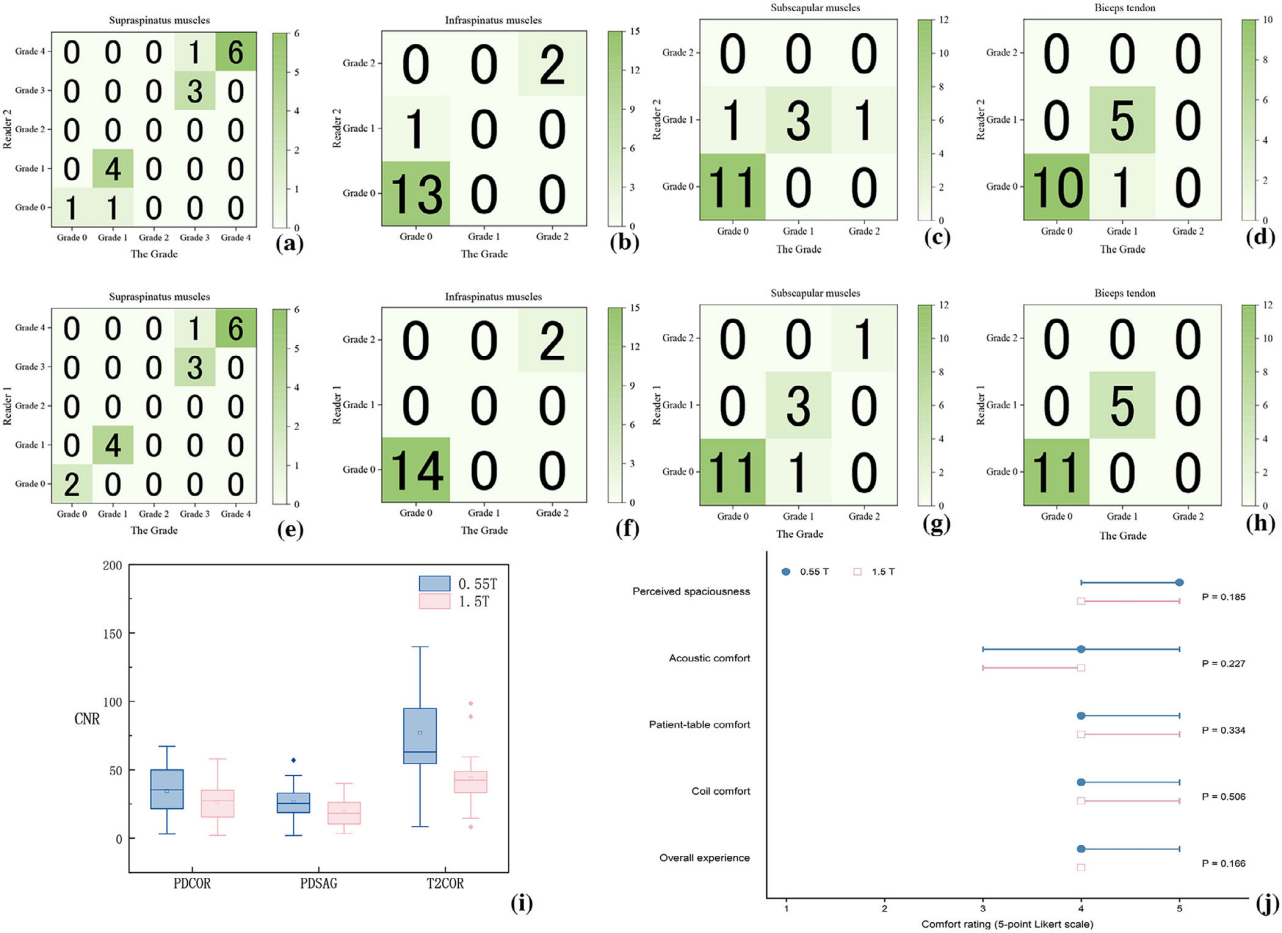
Statistical plots of lesion assessments, CNR and comfort scores in comparison between 0.5T and 1.5T. Agreements for lesion assessments between 0.55T and arthroscopy (a–d) were shown for senior reader. Agreements for lesion assessments between 0.55T and arthroscopy (e–h) were shown for junior reader. Box plot (i) in CNR between 0.55T and 1.5T were shown for COR PDWI, COR T2WI and SAG PDWI. Box‐and‐whisker plots of CNR at 0.55 T and 1.5 T. Boxes indicate the interquartile range, horizontal lines indicate medians, square symbols indicate means, and diamond symbols indicate outliers. Whiskers extend to the most extreme values within 1.5 times the interquartile range. Comparison of comfort feeling were shown (j) in all categories. Volunteer comfort ratings for 0.55‐T and 1.5‐T MRI. Markers indicate median scores, and horizontal error bars indicate interquartile ranges (25th–75th percentiles). P values were obtained using paired two‐sided Wilcoxon signed‐rank tests.

**FIGURE 4:**
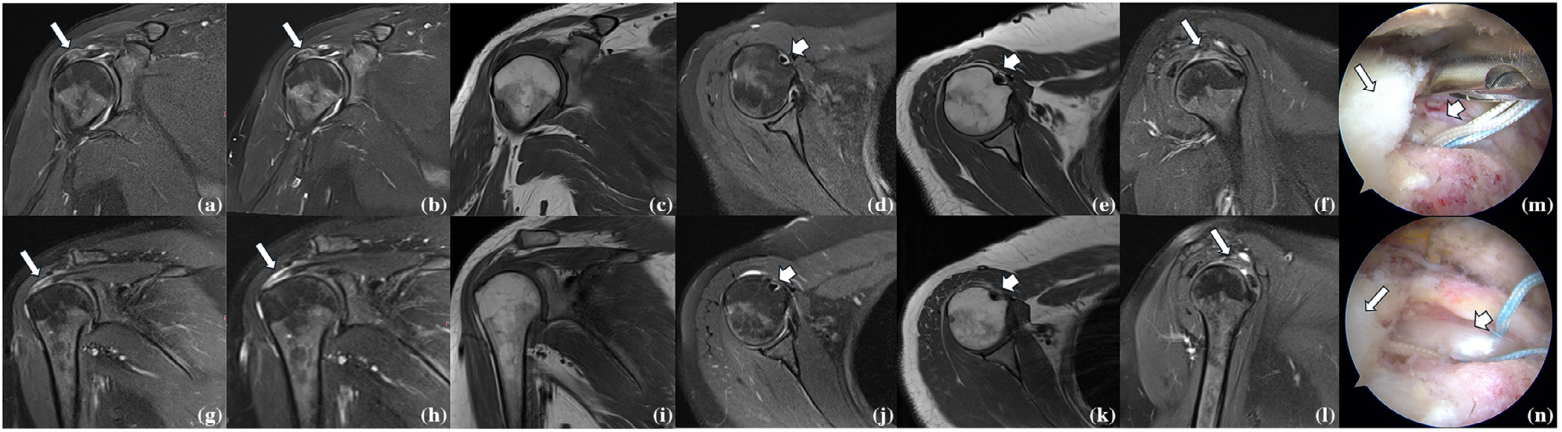
Lesion comparison between 0.55T and 1.5T MRI in a patient. Shoulder MRI of a 52‐year‐old female patient. Images (a–f) show 0.55T scans: coronal (COR) fat‐suppressed proton density‐weighted imaging (fsPDWI), COR fat‐suppressed T2‐weighted imaging (fsT2WI), COR T1WI, axial (AX) fsPDWI, AX T1‐weighted imaging (T1WI), and sagittal (SAG) fsPDWI, respectively. Images G–L show corresponding sequences acquired at 1.5T, matched to (a–f). Images M and N show arthroscopic findings. The long arrow indicates a supraspinatus tendon tear; the short arrow indicates an injury to the long head of the biceps tendon. Both lesions were confirmed by arthroscopy.

**FIGURE 5:**
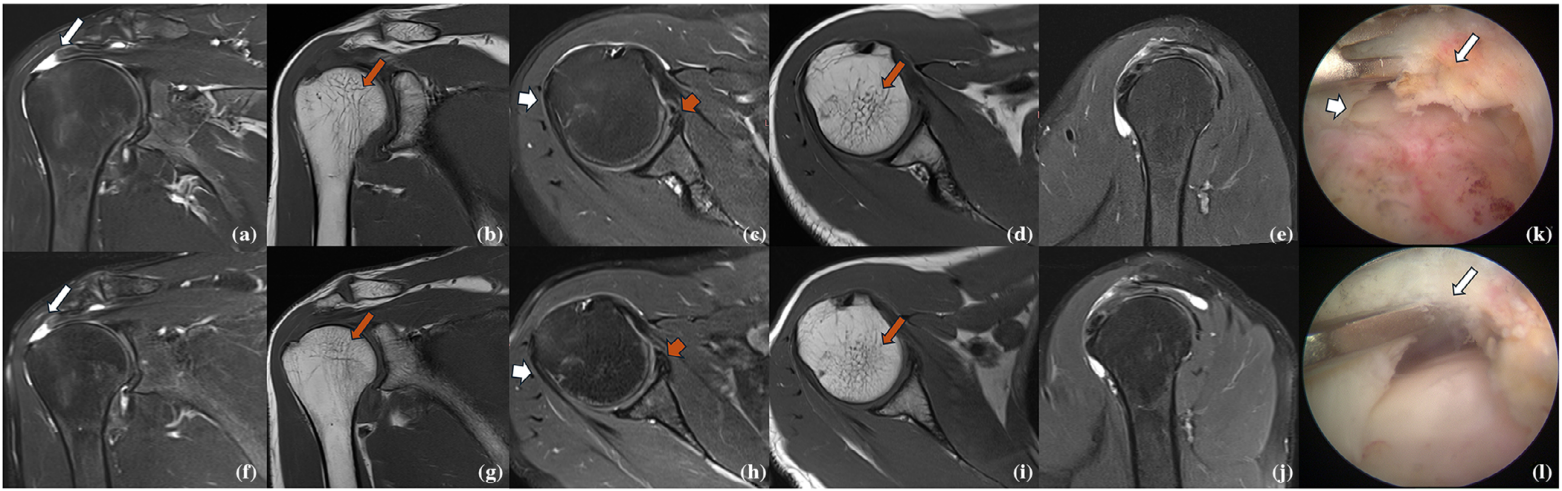
Lesion comparison between 0.55T and 1.5T MRI in a patient. Shoulder MRI of a 48‐year‐old female patient. Images (a–e) show 0.55T scans: coronal (COR) fat‐suppressed T2‐weighted imaging (fsT2WI), COR T1‐weighted imaging (T1WI), axial (AX) fs‐density‐weighted imaging (fsPDWI), AX T1WI, and sagittal (SAG) fsPDWI, respectively. Images (f–j) show corresponding sequences acquired at 1.5T, matched to (a–e). Images k and l show arthroscopic findings. The long white arrow indicates a supraspinatus tendon tear; the short white arrow indicates an infraspinatus tendon injury. Both lesions were confirmed by arthroscopy. Additionally, the long red arrow highlights improved visualization of bone trabeculae at 0.55T (b and d) compared with 1.5T (g and i) on T1WI. The short red arrow indicates degeneration of the glenoid labrum.

**FIGURE 6:**
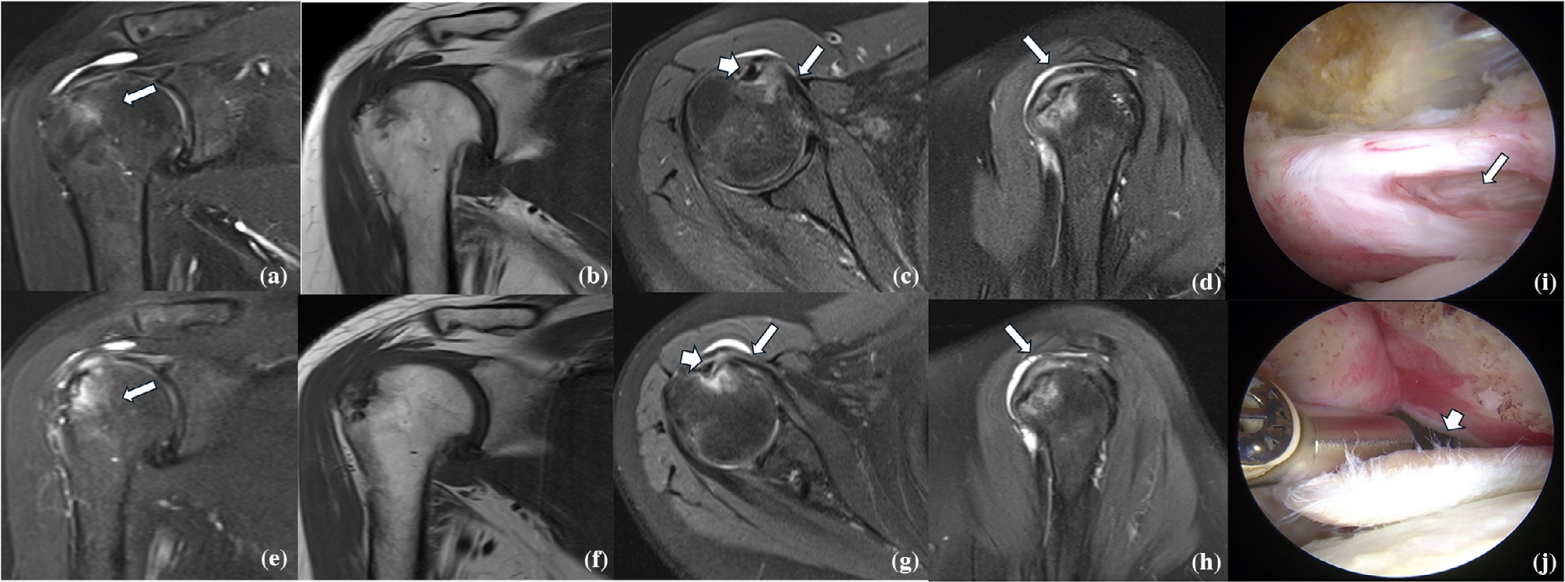
Lesion comparison between 0.55T and 1.5T in a patient. Shoulder MRI of a 63‐year‐old female patient. Images (a–d) show 0.55T scans: coronal (COR) fat‐suppressed T2WI (fsT2WI), COR T1‐weighted imaging (T1WI), axial (AX) fat‐suppressed proton density‐weighted imaging (fsPDWI)fsPDWI, and sagittal (SAG) fsPDWI, respectively. Images (e–h) show corresponding sequences acquired at 1.5T, matched to (a–d). Images I and J show arthroscopic findings. The long arrow indicates bone marrow edema in a and e. The supraspinatus tendon appears normal, a finding confirmed by arthroscopy (short arrow in j). The long arrow also marks a tear of the subscapularis tendon in c, d, g and h, confirmed by arthroscopy (long arrow in i). The short arrow denotes a mild injury of the long head of the biceps‐tendon (c and g).

### 3.5 Diagnostic performance with arthroscopy as reference

A total of 16 patients underwent arthroscopy. The reasons for the other 9 patients without arthroscopy were: (1) 6 of them had slight injury of rotator cuff and chose consecutive treatment; (2) 2 of them chose to observe due to the surgery arrange conflict with other affairs. (3) 1 of them had brain infarction recently.

Perfect interstudy agreement between 0.55T and 1.5T was observed for both readers (Kappa = 1, *P *< 0.001). Agreement between MRI findings and arthroscopic results was perfect for senior reader, who had more diagnostic experience, and good to perfect for junior reader, who had less experience (Table 4; Figures 3, 4, 5, 6).

**TABLE 4:** Interobserver agreement for lesion evaluation and interstudy agreement between 0.55T and 1.5T MRI for each observer.

	Senior Reader				Junior Reader			
	Interstudy agreement between 0.55T and 1.5T		MR‐arthroscopy		Interstudy agreement between 0.55T and 1.5T		MR‐ arthroscopy	
Lesion location	Kappa	P	Kappa	P	Kappa	P	Kappa	P
Bone marrow	1.00	<0.001^*^	–		1.00	<0.001^*^	–	
Glenoid labrum	1.00	<0.001^*^	1.00	<0.001^*^	1.00	<0.001^*^	0.789	<0.001^*^
Supraspinatus muscle tendon	1.00	<0.001^*^	0.947	<0.001^*^	1.00	<0.001^*^	0.889	<0.001^*^
Infraspinatus muscle tendon	1.00	<0.001^*^	1	<0.001^*^	1.00	<0.001^*^	0.871	<0.001^*^
Biceps tendon	1.00	<0.001^*^	1	<0.001^*^	1.00	<0.001^*^	0.862	<0.001^*^
Subscapular muscle tendon	1.00	<0.001^*^	0.881	<0.001^*^	1.00	<0.001^*^	0.733	<0.001^*^
Glenohumeral ligament	1.00	<0.001^*^	–		1.00	<0.001^*^	–	
Coracoacromial ligament	1.00	<0.001^*^			1.00	<0.001^*^		
Coracoclavicular ligament	1.00	<0.001^*^			1.00	<0.001^*^		
Coracohumeral ligament	1.00	<0.001^*^			1.00	<0.001^*^		
Joint effusion	1.00	<0.001^*^			1.00	<0.001^*^		

* Statistically significant *P*‐value.

### 3.6 Volunteer comfort survey

Although no statistically significant differences were observed in comfort ratings between 0.55T and 1.5T, the 0.55T system consistently received higher average scores across all evaluated categories (Figure 3).

## 4 DISCUSSION

The results of this study demonstrated that parallel imaging combined with DL reconstruction at 0.55T realized the image quality that was comparable to that of traditional 1.5T MRI, without increasing scan time. When referenced against arthroscopic findings, both MRI systems showed high consistency in diagnostic performance for rotator cuff injuries.

Low‐field MRI systems with larger bore sizes and reduced metal artifacts have gained increasing popularity, particularly among patients with obesity, pregnancy, or a recent history of surgery.^5^, ^6^, ^7^, ^8^, ^9^, ^10^ Nevertheless, limitations such as relatively longer scan times and lower SNR compared with high‐field systems have restricted broader clinical adoption. With advancements in coil technology and novel reconstruction methods, such as DL algorithms, the 0.55T system has gained increasing clinical adoption—the present study revealed robust potential in shoulder imaging. There reported a high morbidity rate of shoulder disorders in the general population.^20^, ^21^ Therefore, further investigation and application of shoulder MRI at 0.55T with DL are expected to meet clinical demands.

Imaging assessments demonstrated that 0.55T with DL produced image quality comparable to, and in some cases better than, that of 1.5T without DL. A previous study showed similar image quality in knee imaging, although inferior performance at 0.55T was noted in fsPDWI and fsT2WI sequences.^4^ In contrast, the present study revealed higher image quality and improved SNR in nearly all sequences, particularly for T1WI and bone marrow SNR, which was in accordance with the phantom study (Figure S‐1). These improvements may be attributed to the enhanced performance of the contour coil and the application of DL reconstruction methods. In addition, the application of deep learning showed greater influence on T1WI than T2WI and PDWI.

Inherent to the 0.55 T system itself, its native spatial resolution is inevitably inferior to that of a 1.5 T scanner. Nevertheless, we implemented two optimization strategies: upgraded coil hardware and fine‐tuned sequence parameters—including prolonged TR and reduced FOV. These adjustments allow us to match the scan time of conventional 1.5 T protocols while preserving spatial resolution to the greatest extent possible. Furthermore, DL reconstruction further facilitates this goal.

Previous study has reported the artifacts shown in the DL‐reconstructed images. The motion artifacts would be brought due to a relatively long scanning time.^22^ Meanwhile, they also reported the milder urine flow artifacts with DL‐half‐Fourier single‐shot turbo spin echo sequence (HASTE) in comparison to standard HASTE, and they speculated that it might be attributed to its rapid acquisition scheme. As so far, there were little such artifacts shown in DL‐images in our study. However, lower magnetic field also brought lower SNR of marginal areas such as brachial plexus (Figure 2d, Figure 4d). Therefore, patients with suspected tumor would be more suitable for 1.5T with large scale.

Lesion assessment at 0.55T with DL demonstrated high concordance with both 1.5T and arthroscopy findings, a comparison not previously reported in studies involving 0.55T. According to existing literature, the sensitivity and specificity of shoulder MRI in detecting rotator cuff injuries range from 85% to 95% when referenced against arthroscopy.^23^, ^24^, ^25^ The present study yielded comparable results at both 0.55T with DL and traditional 1.5T. High interstudy agreement is consistent with the published sensitivity and specificity of previous studies.^23^, ^24^, ^25^ Greater diagnostic consistency was achieved by the senior radiologist, likely due to a longer duration of clinical experience. However, the agreements between junior reader and arthroscopy did not show high consistency. We speculated that the main reason could be the insufficient experience, because the interstudy agreements were high. Nevertheless, more readers with different experience need be enrolled for further investigation.

Numerous studies have investigated the clinical application of lower‐field MRI;^1^, ^2^, ^3^, ^4^, ^5^, ^6^, ^7^, ^8^, ^9^, ^10^ however, most have focused on reducing the magnetic field strength from 1.5T. The MAGNETOM Free Max 0.55T system, with its open 80‐cm bore, offers enhanced patient comfort and accessibility, particularly for individuals with obesity or claustrophobia. Thilo Rusche et al evaluated patient comfort between the MAGNETOM Free Max and a 1.5T system (Avanto Fit, Siemens Healthineers) with a 60‐cm bore.^10^ Their results indicated that patients perceived 0.55T MRI as more comfortable than conventional 1.5T systems, including examinations of the head, spine, hip, and extremities. In the present study, shoulder imaging comfort was compared for the first time between the 0.55T system and a 1.5T system (Aera, Siemens Healthineers) with a 70‐cm bore. Although the differences were not statistically significant, the 0.55T system consistently received relatively higher average scores. Comfortable imaging experiences are increasingly important in MRI, since they could help to reduced patient anxiety and improved overall well‐being during examinations. However, only healthy volunteers have been surveyed for comfort feeling because the intervals between the two examinations were between 10–14 days. To avoid bias, comfort surveys have not been conducted in patients’ group (they all finished in one day). But further experiments should be investigated among patients. In addition, since body composition might also have the influence on SNR, further investigation with different body mass index (BMI) could be also worth studying.

Another advantage of the larger bore size is the ability to accommodate more flexible patient positioning, such as abduction external rotation, which improves visualization of potentially occult Perthes labral tears. The larger bore diameter of the 0.55 T scanner provides extra spatial margin around patients, enabling more flexible and accurate isocenter positioning compared with the conventional 1.5 T system. For patients with broad shoulder girth, bulky body habitus or limited mobility, the wider bore helps avoid forced compensatory postures and facilitates centering the shoulder joint at the scanner's isocenter, which further improves imaging uniformity and signal consistency across the field of view. The current study confirmed the feasibility of shoulder imaging at 0.55T in an abducted position of 45°. These findings suggest that the MAGNETOM Free Max system holds considerable promise for broader clinical application in the future. However, patients with rotator cuff injury were not suitable for the additional position. Therefore, the flexible positions have not been conducted in the patients’ cohort.

Recent advances have introduced novel methods with the potential to enhance the entire MRI workflow,^16^, ^26^, ^27^ including image reconstruction, interpretation, and quantitative analysis. For image reconstruction, DL networks are often utilized during a training process to establish optimal transformation between input and target data by minimizing a defined loss function. Several studies have investigated the feasibility of DL applications in this context.^28^, ^29^, ^30^ Chen et al. et al. demonstrated that CNN‐based reconstruction techniques possess strong potential for noise suppression, structural preservation, and lesion detection.^29^ Schlemper et al. evaluated the performance of a CNN‐based approach in reconstructing 2D cardiac MRI; they reported decreased reconstruction error and increased reconstruction speed.^30^ In 2023, our study confirmed the clinical feasibility and superiority of a CNN‐based algorithm revealing significant improvements in acquisition time, image quality of shoulder imaging at 3T.^14^ In the present study, DL‐MRI improved image quality and reduced acquisition time in PDWI and T2WI for shoulder imaging.

In present study, we chose the routine shoulder scan protocol including 5 sequences in our hospital. Among the 5 sequences, the most frequently used sequences including fsPDW, fsT2WI and T1WI were be covered. Usually, 3 sequences with fsPDW or fsT2WI in three directions and 1 sequence of T1WI could be the routine protocol. However, PDWI would have better SNR of ligaments or muscle tendon, but fsT2WI would have less magic angle effect. Therefore, we would like to add one sequence of fsT2WI in routine work. The total scanning time would be less if only 4 sequences have been applied.

Some limitations should be acknowledged. First, only patients with suspected rotator cuff injuries were included in this investigation, and only 2 readers gave the lesion assessment. Future studies should involve a broader spectrum of shoulder pathologies and more readers’ evaluation. Meanwhile, since the age gap between healthy volunteers and patients was substantial, participants with more age group should be investigated. Second, MR arthrography with direct intra‐articular contrast administration should be considered to improve labral tear evaluation.^31^, ^32^ Third, the images comparison just showed 0.55T with DL reconstruction could have comparable value in clinical application with conventional 1.5T. Further study might have been tried to conduct the comparison to the 1.5T with DL. Finally, we haven't conducted the comparison to other MR systems or 3.0T which are widely used in the clinical setting now, and we believe that the further study would bring more scientific value.

## 5 CONCLUSION

The 0.55T MRI system, equipped with contour coils and optimized DL‐based sequences, demonstrated image quality that was comparable to or better than that of the traditional 1.5T system, without increasing scan time in both young volunteers and patients with rotator cuff injury. The open 80‐cm bore of the 0.55T MRI system allowed greater flexibility in positioning.

## AUTHOR CONTRIBUTIONS

**Jing Liu**: Conceptualization; data curation; formal analysis; investigation; writing—original draft. **Yue Gao**: Data curation; formal analysis; validation; writing—original draft. **Ge Gao**: Data curation; formal analysis; validation; writing—original draft. **Wei Li**: Conceptualization; data curation; formal analysis; methodology; validation. **Xiang Wang**: Data curation; investigation; methodology. **Jinxia Zhu**: Conceptualization; data curation; formal analysis; methodology. **Xiaoming Liu**: Data curation; formal analysis; writing—review & editing. **Xiaoye Wang**: Data curation; investigation. **Kun Zhou**: Methodology; resources; software. **Heng Liu**: Methodology; supervision; writing—review & editing. **Jianxing Qiu**: Conceptualization; data curation; investigation; supervision.

## CONFLICT OF INTEREST STATEMENT

Four authors (Jinxia Zhu, Xaoming Liu, Xiaoye Wang, Kun Zhou) are employees of Siemens Healthcare. Otherwise, the authors of this manuscript declare no relationships with any companies whose products or services may be related to the subject matter of the article.

## FUNDING STATEMENT

Beijing Municipal Science and Technology Commission Capital Clinical Characteristic Application Research (Z181100001718057/ Heng Liu) National High Level Hospital Clinical Research Funding (Interdepartmental Clinical Research Project of Peking University First Hospital) (2023IR26/Jing Liu)

## ETHICS STATEMENT

This prospective, single‐center study was conducted in accordance with the Declaration of Helsinki and was approved by the institutional review board of Peking University First Hospital (protocol code‐2024‐007). Written informed consent was obtained from all participants, including both volunteers and patients.

## Supporting information



Supporting Information

Supporting Information

Supporting Information

Supporting Information

Supporting Information

## Data Availability

Authors will share data upon request to the corresponding author.
