# Supplementary material for: Diagnostic image quality of shoulder magnetic resonance imaging at 0.55T in healthy subjects and patients with rotator cuff injury: Prospective comparison with 1.5T
Source: J Appl Clin Med Phys. 2026 Oct 20;27(11):e70825. doi: 10.1002/acm2.70825 (PMC13682617; doi:10.1002/acm2.70825)
Supplement: Supplementary file 1 [file ACM2-27-e70825-s001.docx]

**Supplementary 3	MRI parameters for phantom study**

|  | Coronal T1WI | | | | Coronal fsT2WI | | | | Coronal fsPDWI | | | |
| --- | --- | --- | --- | --- | --- | --- | --- | --- | --- | --- | --- | --- |
| Figure S2 | A | D | G | J | B | E | H | K | C | F | I | L |
| Repetition time  (ms) | 640 | 500 | 500 | 640 | 2100 | 5000 | 5000 | 2100 | 2100 | 3200 | 3200 | 2100 |
| Echo time (ms) | 11 | 12 | 12 | 11 | 58 | 69 | 69 | 58 | 36 | 42 | 42 | 36 |
| Field of view  (mm^2^) | 180*225 | 160*200 | 160*200 | 180*225 | 180*225 | 160*200 | 160*200 | 180*225 | 180*225 | 160*200 | 160*200 | 180*225 |
| Slice thickness  (mm) | 4 | | | | 4 | | | | 4 | | | |
| Slice gap (mm) | 1 | | | | 1 | | | | 1 | | | |
| Acquisition matrix | 320*240 | 224*180 | 224*180 | 320*240 | 320*224 | 192*154 | 192*154 | 320*224 | 320*224 | 208*166 | 208*166 | 320*224 |
| Reconstructed matrix | 640*640 | 448*448 | 448*448 | 640*640 | 256*256 | 384*384 | 384*384 | 256*256 | 320*320 | 416*416 | 416*416 | 320*320 |
| Phase oversampling | 80% | 200% | 200% | 80% | 100% | 200% | 200% | 100% | 100% | 200% | 200% | 100% |
| Echo train length | 3 | 3 | 3 | 3 | 8 | 12 | 12 | 8 | 8 | 6 | 6 | 8 |
| Deep Resolve Boost with Deep Resolve sharp | —— | High | —— | High | —— | High | —— | High | —— | High | —— | High |
| Acceleration factors/mode | —— | 3/GRAPPA | —— | 3/GRAPPA | —— | 4/GRAPPA | —— | 4/GRAPPA | —— | 3/GRAPPA | —— | 3/GRAPPA |
| Number of excitations | 1 | 2 | 2 | 2 | 1 | 2 | 2 | 2 | 1 | 1 | 1 | 1 |
| Number of slices | 16 | | | | 16 | | | | 16 | | | |

Abbreviations: PDWI, proton density-weighted imaging; T1WI, T1-weighted imaging; T2WI, T2-weighted imaging.
