# Supplementary material for: Diagnostic image quality of shoulder magnetic resonance imaging at 0.55T in healthy subjects and patients with rotator cuff injury: Prospective comparison with 1.5T
Source: J Appl Clin Med Phys. 2026 Oct 20;27(11):e70825. doi: 10.1002/acm2.70825 (PMC13682617; doi:10.1002/acm2.70825)
Supplement: Supplementary file 2 [file ACM2-27-e70825-s002.docx]

**Supplementary 2	Phantom experiments at 1.5T and 0.55T**

A round region of interest (ROI) with an area of approximately 200 mm^2^ was placed in the center of phantom on the middle slice for each sequence, to measure the signal intensity (SI) of phantom. Meanwhile, a ROI with an area of approximately 50 mm^2^ was placed in air distant from the phantom, to determine the standard deviation (SD) of background noise on the same slice for each sequence. Therefor, SNR was calculated by SI/SD.

Figure S1 A-C stands for phantom imaging for T1WI, fsT2WI and PDWI at 1.5T, and the SNRs were 159.51, 102.56 and 91.58, respectively.

D-F stands for phantom imaging for T1WI, fsT2WI and PDWI at 0.55T with deep learning, and the SNRs were 339.73, 127.04 and 95.33, respectively.

G-I stands for phantom imaging for T1WI, fsT2WI and PDWI at 0.55T without deep learning, and the SNRs were 181.1, 123.7 and 109.75, respectively.

J-L stands for phantom imaging for T1WI, fsT2WI and PDWI at 0.55T with deep learning, and also with the same parameters as 1.5T, and the SNRs were 153.26, 177.14 and 97.11, respectively.

The parameters for each sequence were shown in Supplementary 3.
